# Psychological health status of Chinese university students: based on Psychological Resilience Dynamic System Model

**DOI:** 10.3389/fpubh.2024.1382217

**Published:** 2024-05-23

**Authors:** Junqiang Fan, Yuxin Huang, Fei Yang, Yongjie Cheng, Jingjing Yu

**Affiliations:** ^1^School of Economics and Management, Zhejiang University of Science and Technology, Hangzhou, Zhejiang, China; ^2^School of Environment and Natural Resources, Zhejiang University of Science and Technology, Hangzhou, Zhejiang, China; ^3^Hangzhou Xixi Hospital, Hangzhou, Zhejiang, China

**Keywords:** Chinese university students, mental health, psychological resilience, self-efficacy, social support

## Abstract

**Introduction:**

The mental health of unverisity students is influenced by diverse factorsis multifaceted, requiring further investigation to evaluate its current status and determinants. The present study aims to address this gap by targeting Chinese university students and employing the Psychological Resilience Dynamic System model. Through a questionnaire survey, this research endeavors to explore the mental health status and influencing factors. Ultimately, the findings of this study aim to provide a theoretical basis and tailored practical guidance for the development of mental health intervention strategies for university students.

**Methods:**

Based on the Psychological Resilience Dynamic System Model, the mental health status of 3,390 Chinese university students from 15 universities was empirically investigated with the principle of stratified sampling and the geographical distribution and disciplinary diversity of universities. The questionnaires used included Kessler psychological distress scale, psychological resilience scale,positive psychological capital scale, family hardiness index and social support scale. Among the participants, 47.85% were male and 52.15% were female. Regarding the origin, 42.89% of the students were from rural areas, while 57.11% were from urban areas.

**Results:**

Key findings unveil: (1) A prevalence of 24.54% in students has suboptimal mental health, with 18.70 and 5.84%, respectively, representing those with poor and relatively poor mental health conditions; (2) A noteworthy negative correlation (*p* < 0.01) between mental health scores of university students and nine pivotal factors, including psychological resilience, self-efficacy, optimism, hope, resilience, family resilience, objective support, subjective support, and support utilization; (3) Eight factors, including grade, family economic status, psychological resilience, self-efficacy, optimism, family resilience, objective support, and support utilization, emerge as significant predictors of university students’ mental health (*p* <0.001), collectively elucidating 57.9% of the total variance in mental health.

**Discussion:**

The aforementioned research results, indicate that the influencing factors on the mental health of university students encompass four main aspects. These include individual demographic factors such as grade and family economic status, positive psychological capital factors such as psychological resilience, self-efficacy, optimism, hope, and resilience, family resilience factors including responsibility, control, and challenge, and societal support factors including objective support, subjective support, and support utilization. Based on this, this paper focuses on four recommendations: giving full play to the leading role of universities in mental health education and stress intervention, strengthening the educational power of positive family ideals and role modeling, building a support system for positive social atmosphere and psychological counseling, and improving the self-shaping ability of university students’ psychological resilience and positive psychological capital. These recommendations aspire to better promote the mental health of university students and provide a strength reserve for psychological problem intervention.

## 1 Introduction

### 1.1 Research background

In 2023, the landscape of Chinese higher education enrollment has surpassed 46 million students, with university graduates numbering 11.58 million, indicating year-on-year growth. The phase of rapid physical and psychological development experienced by university students often coincides with heightened stress and adversity, giving rise to mental health challenges (1). Particularly salient is the profound impact of the COVID-19 pandemic (2), representing an unparalleled public health emergency in China in terms of speed of transmission, breadth of influence, and complexity of control efforts since the establishment of the People’s Republic of China (3). This pandemic not only jeopardizes human health but also precipitates psychological distress and a sense of helplessness, bringing widespread social and economic pressures. In this context, university students contend with significant employment pressures, inherently fostering psychological anxiety and pessimism. Consequently, this scenario poses a formidable challenge to psychological health education within Chinese higher education institutions. The cultivation of a positive and robust psychological state among university students emerges as a paramount imperative for educational institutions. A nuanced comprehension of the prevailing psychological well-being and its determinants among university students assumes crucial significance for the development of targeted intervention strategies. This will guide students in confronting challenges with a constructive and optimistic mindset, nurture their mental and physical well-being and further facilitate enhanced adaptability to change, the reconstruction of confidence, and a proactive orientation towards the future. In the post-pandemic era, the mental health of college students is influenced by various factors, including both internal and external elements, as well as their interactions. However, no studies were found in current research that specifically investigated the mental health status of Chinese university students using the Psychological Resilience Dynamic System Model, which incorporates individual trait system derived from psychological resilience as internal protective factors, and family and social support system as external protective factors. Therefore, this study focuses on Chinese university students in the post-pandemic era, examining their mental health from four perspectives: psychological resilience, individual trait system (self-efficacy, optimism, hope, and resilience), family support system measured by family resilience, and social support system measured by social support, with the first two as measures of internal protective factors and the latter two systems as measures of external protective factors. By integrating both internal and external factors that influence students’ mental health, this study aims to analyze the current mental health status of Chinese university students and its influencing factors. Additionally, it aims to explore how the theoretical framework of the Psychological Resilience Dynamic System Model can be utilized to formulate interventions that promote mental health. This research intends to guide mental health educators in universities on how to better address the mental health status of college students, promote their well-being, and provide theoretical foundations for future longitudinal studies by offering appropriate psychological counseling to college students.

### 1.2 The correlation between psychological resilience and mental health

The term “psychological resilience,” originally denoting concepts of “rebound” and “recovery,” is frequently translated by Chinese scholars as “resilience” or “restorative power.” Subsequently, researchers have increasingly embraced the term “psychological resilience” in their investigations (4). This conceptual framework refers to an individual’s adaptive or resilient capacity when confronted with substantial stress, adversity, trauma, or disaster (5). Psychological resilience serves as a prognosticator of an individual’s mental health status after major stressors and represents a decisive factor in the successful recovery from stress coping efforts (6). Influencing factors on psychological resilience span both intrinsic dimensions, such as individual psychological capital and coping behaviors, and extrinsic dimensions such as family and society. According to Stewart et al.’s psychological resilience process model, individuals establish a psychological protective mechanism when faced with stress, dynamically augmenting adaptability by reconfiguring the relationship between the individual and the surrounding environment (7). The influencing factors of mental health are the result of the interaction of various factors. The main factors affecting individual mental health can be divided into environmental factors such as family, community, and social factors, and individual factors such as physiological and psychological factors (disease, self-awareness, and stress coping, etc.). Psychological resilience can predict the level of an individual’s mental health after encountering stress, and is not only closely related to mental health but also an important indicator thereof. Huang et al. (8) explored the relationship between Chinese thinking patterns and mental health: the role of psychological resilience and self-esteem. The results showed that psychological resilience played a mediating role between thinking patterns and mental health. Lu et al. (9) investigated the impact of residents’ emotional regulation on mental health during sudden public health emergencies, and found that psychological resilience played a fully mediating role between cognitive reappraisal and mental health status (with an effect value of 92.86%), and a masking effect between expressive suppression and mental health status (with an effect value of 47.2%). Cultivating good psychological resilience in individuals can help maintain relatively stable and healthy psychological states when facing sudden public health emergencies. Sun et al. (10) explored the relationship between college students’ psychological health, psychological resilience, coping strategies after major negative life events, and found that psychological resilience not only directly affects the mental health of college students, but also indirectly affects it through coping strategies, with gender moderating the relationship between psychological resilience and mature coping styles.

### 1.3 Theoretical model of psychological resilience: the Psychological Resilience Dynamic System Model

Leveraging global and domestic research, Chinese scholar Zheng (11) synthesized various factors that influences psychological resilience into a dynamic system model. This model encompasses individual trait system, family environment system, and social support system. The individual trait system encompasses elements such as self-efficacy, positive psychological capital, and psychological defense mechanisms. The family environment system considers factors such as family composition, economic status, and parenting styles, while the social support system encompasses social support from schools, peers, and communities. Zhang (12) systematically investigated the dynamic system model of psychological resilience in post-coronary artery bypass grafting patients, identifying factors including self-efficacy, personality traits, coping styles, family resilience, and social support. Elements within the psychological resilience dynamic system model can be broadly classified into protective and risk factors. Protective factors function to shield individuals from the adverse effects of risk factors and play a decisive role in fostering psychological resilience (13). Multiple studies (14–16) have indicated that the internal protective factors of psychological resilience accompany an individual’s psychological qualities throughout their growth process. As individual trait system possess characteristics of initiative, intervention, continuity, and stability, they play a decisive role within the entire dynamic system. External protective factors such as the family environment and social support system play crucial roles in the physical and mental development of individuals. Discordant family relationships can lead to adverse emotions such as anxiety, withdrawal, and tension in individuals. Social forces such as friends, relatives, and groups not only provide assistance in various material and spiritual aspects but also alleviate the adverse consequences of events such as stress and threats, thereby enhancing the individual’s level of psychological resilience.

Grounded in this theoretical framework, the current study employs the psychological resilience dynamic system model as its foundation. Through empirical research, this paper explores the influencing factors affecting the mental health of Chinese college students in the post-pandemic era from the four perspectives of psychological resilience, individual trait system, family resilience and social support, and excavates the protective factors of college students’ mental health, in order to boost strength to cope with psychological challenges, and provide theoretical basis and personalized practical guidance for the formulation of college students’ mental health interventions in the next step, which has good theoretical significance and practical value.

## 2 Methods

### 2.1 Research participants

The research participants are current university students enrolled in universities in Zhejiang Province, China. Zhejiang Province is renowned for its economic prosperity, attracting students from diverse regions across China to pursue higher education within its institutions. As a microcosm of national higher education, Zhejiang’s tertiary institutions reflect the comprehensive standards and quality prevalent throughout the country. This demographic provides a rich and diverse pool of participants, ensuring that findings derived from this sample are broadly representative of the broader landscape of higher education in China. As of 2023, there are a total of 60 undergraduate universities in Zhejiang Province. These universities range from prestigious “Double First-Class” institutions such as Zhejiang University to regular undergraduate universities like Zhejiang University of Technology, as well as local university such as Shaoxing University. Following the principles of stratified sampling and considering both the geographical and disciplinary distribution of these institutions, a sample of 15 undergraduate universities was selected, which represents a diverse cross-section of higher education institutions in China, including Zhejiang University, Zhejiang University of Technology, Zhejiang Normal University, Ningbo University, Zhejiang Sci-Tech University, Hangzhou Dianzi University, Zhejiang Gongshang University, China Jiliang University, Zhejiang Chinese Medical University, Wenzhou Medical University, Hangzhou Normal University, Zhejiang University of Science and Technology, Shaoxing University, Huzhou University, and Hangzhou City University. From October 1 to October 31, 2023, a questionnaire survey using an online survey platform named Wenjuanxing was conducted among the university students in these 15 universities. Inclusion criteria stipulated that participants must be currently enrolled students at the selected universities, fully informed about the research objectives, willing to participate voluntarily, able to cooperate in completing the survey, and had resided within China in the 8 weeks preceding the survey. Exclusion criteria included individuals with significant physical illnesses or recent psychological traumas, such as recent serious illness or death of family members. Wenjuanxing implemented restrictions on the number of responses per IP address, allowing only one response per IP. A total of 3,536 questionnaires were collected, and after excluding incomplete responses, 3,390 valid questionnaires were obtained, resulting in an effective response rate of 95.87%.

### 2.2 Research instruments

#### 2.2.1 Demographic questionnaire

A self-designed questionnaire encompasses information such as the university students’ grade, gender, age, major, hometown, and family economic status. The classification of family economic status distinguishes between economically disadvantaged students and non-economically disadvantaged students. The criteria for this classification are based on the results of the identification of economically disadvantaged students for the academic year 2023–2024 by universities and the registration record in the national poverty alleviation system.

#### 2.2.2 Kessler psychological distress scale (K10)

The K10 (Cronbach’s *α* = 0.871) in Chinese version (17) was adopted to measure the psychological status in the most recent four weeks period. Participants are asked on a 5-point scale from 1 (almost never) to 5 (always). Higher scores indicate poorer psychological conditions, with scores under 15 indicating good mental health, 16 to 21 indicating mild mental disorder, 22 to 29 indicating moderate mental disorder, and ≥ 30 indicating severe mental disorder.

#### 2.2.3 Psychological Resilience Scale

The Chinese version of the Psychological Resilience Scale (Cronbach’s *α* = 0.92) revised by Yu and Zhang (18), comprises 25 items scored on a 5-point scale, with responses ranging from “never” (0 points) to “always” (4 points). The total score can reach 100 points, with higher scores indicating better psychological resilience.

### 2.3 Positive psychological capital scale

The scale was adapted from Zhang et al. (19), including four dimensions with 26 items in total: self-efficacy (7 items), optimism (6 items), hope (6 items), and resilience (7 items), 5 reverse-scored questions and 21 positive-scored questions are rated on a 7-point scale, with higher scores reflecting higher levels of positive psychological capital. The Cronbach’s *α* coefficients for this questionnaire are as follows: self-efficacy 0.825, optimism 0.803, hope 0.770, resilience 0.758, and overall 0.893.

#### 2.3.1 Family hardiness index (FHI)

A scale developed by McCubbin et al. (20), were used in this study with three dimensions: responsibility, control, and challenge, with a total of 20 items, which adopts a 4-point scale from 1 (strongly disagree) to 4 (strongly agree). The total score ranges from 20 to 80, with higher scores indicating better family resilience. The Cronbach’s *α* coefficients for this questionnaire are as follows: responsibility 0.691, control 0.625, challenge 0.534, and overall 0.807.

#### 2.3.2 Social support scale

The scale developed by Xiao (21), including three dimensions: objective support (3 items), subjective support (4 items), and support utilization (3 items), are used in this study with 10 items in total. The overall Cronbach’s *α* coefficient for this scale is 0.92, indicating good reliability and validity. Higher scores in social support or its three dimensions signify a greater level of overall support or support in each dimension.

### 2.4 Implementation and statistical methods

The aforementioned questionnaires constitute an integrated whole and were distributed through the Wenjuanxing platform. The survey was conducted by transferring the questionnaire links to the corresponding grade and class groups through counselors and class advisers at the surveyed 15 universities. After obtaining data from the Wenjuanxing platform, statistical analysis was processed with SPSS 22.0 software. All data underwent dual verification and dual entry by two individuals. Descriptive analyses were conducted for demographic information and mental health indicators, psychological resilience, positive psychological capital, family resilience, and social support status. Single-factor analysis involved two independent sample t-tests and analysis of variance (ANOVA). Pearson correlation analysis was employed to explore relationships between variables. Multiple-factor analysis of the psychological resilience dynamic system model utilized multivariate linear regression analysis.

## 3 Results

### 3.1 Mental health status of university students and univariate analysis

**Figure 1:**
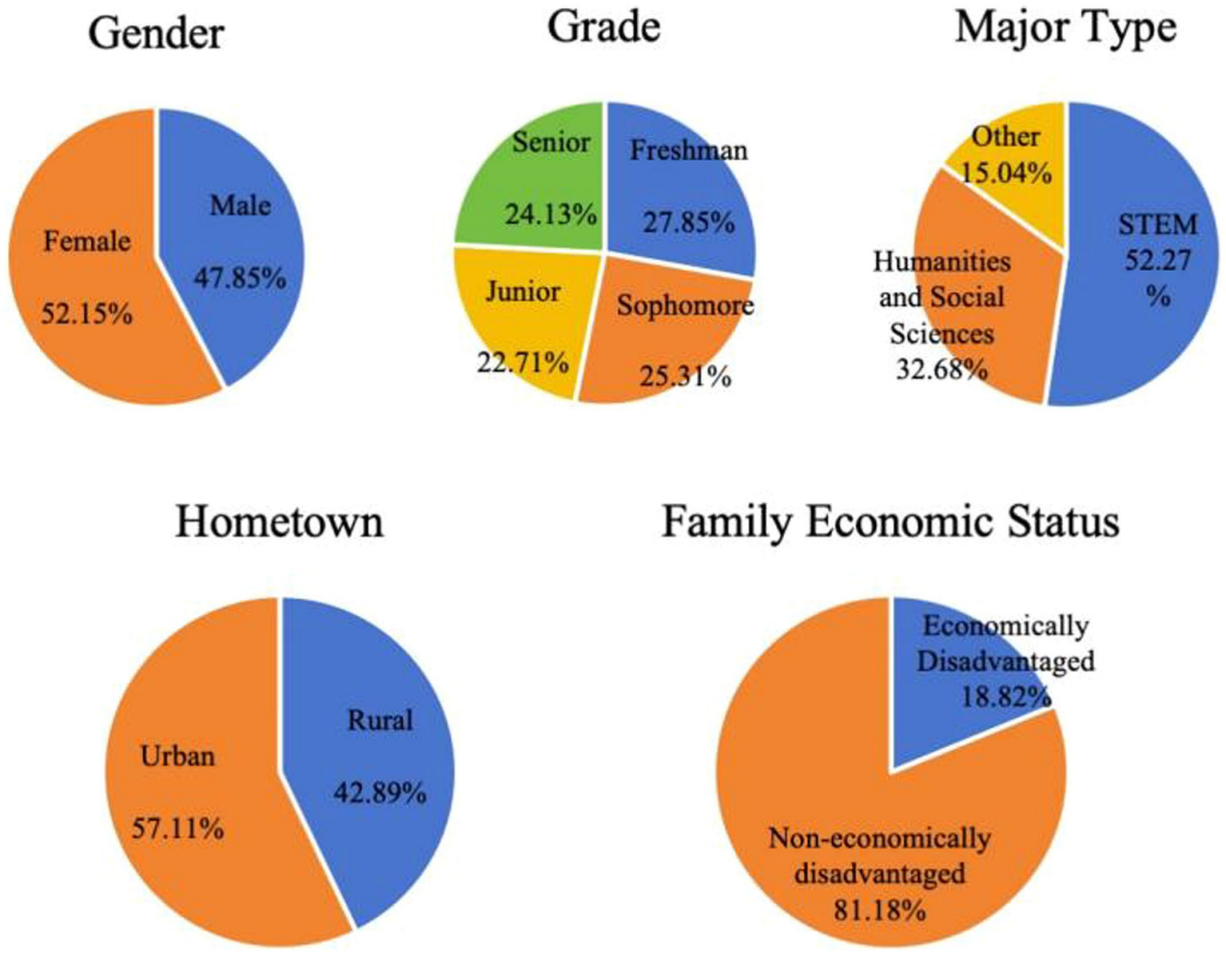
Pie charts of demographic factors proportions.

The survey shows that the average score for the mental health status of 3,390 university students is 19.85 ± 5.17 points. Among them, there were 1,056 university students with good mental health, accounting for 31.15%, 1,502 with average mental health, 44.31%, 634 with poor mental health, 18.70%, and 198 with very poor mental health, accounting for 5.84%. Overall, the total proportion of students facing poor and very poor mental health status is as high as 24.54%, which shows that the current mental health status of university students cannot be ignored. In terms of demographic disparities, statistically significant differences are observed in the mental health status of students across different grades and family economic conditions ( *p* < 0.05). However, variations in mental health status related to gender, major type, and hometown of university students are not significant ( *p* > 0.05). Detailed information is available in Table 1. Proportions based on different demographic factors are illustrated in Figure 1.

**Table 1:** Comparison of mental health scores among university students across different demographic factors (*n* = 3,390).

Demographic factors	Classification	Number of participants	Mental health score	Statistical value	*p*-value
Gender	Male	1,622	18.92 ± 4.85	1.329^*^	0.091
	Female	1768	20.18 ± 5.33		
Grade	Freshman	944	15.80 ± 4.25	14.722^#^	0.000
	Sophomore	858	17.84 ± 4.55		
	Junior	770	21.29 ± 5.42		
	Senior	818	24.85 ± 5.90		
Major Type	STEM	1772	18.89 ± 4.19	0.473^#^	0.174
	Humanities and Social Sciences	1,108	19.64 ± 5.23		
	Other	510	20.60 ± 5.62		
Hometown	Rural	1,454	21.05 ± 5.77	2.153^*^	0.078
	Urban	1935	19.11 ± 4.23		
Family Economic Condition	Economically disadvantaged	638	23.35 ± 5.41	8.527^*^	0.000
	Non-economically disadvantaged	2,752	17.81 ± 4.66		

^*^Represents *t*-values; ^#^ represents *F*-values.

### 3.2 Correlation analysis of mental health with psychological resilience, positive psychological capital, family resilience, social support

**Figure 2:**
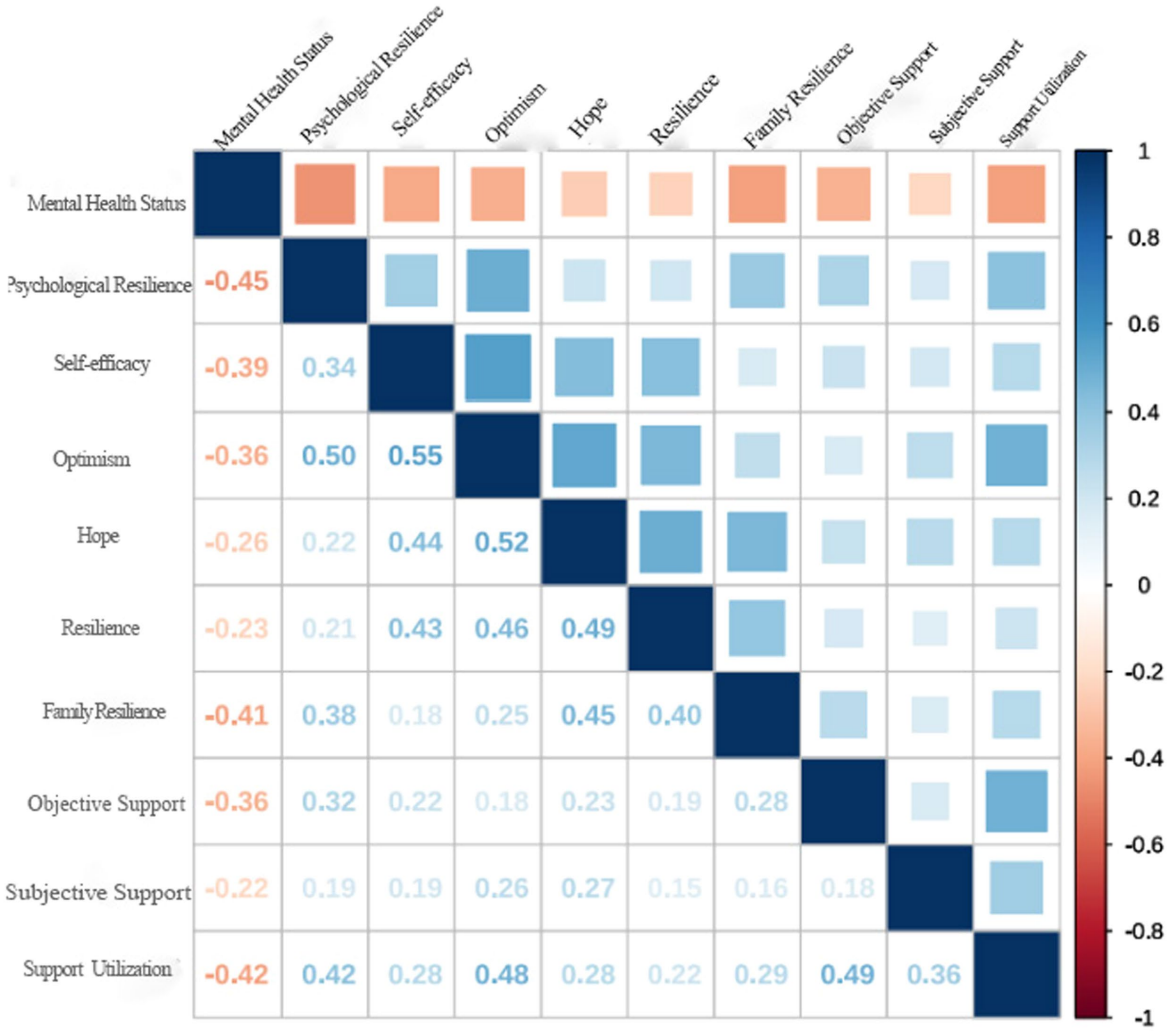
Heat map analysis of the correlation between psychological resilience, psychological capital, family resilience, social support, and mental health.

**Figure 3:**
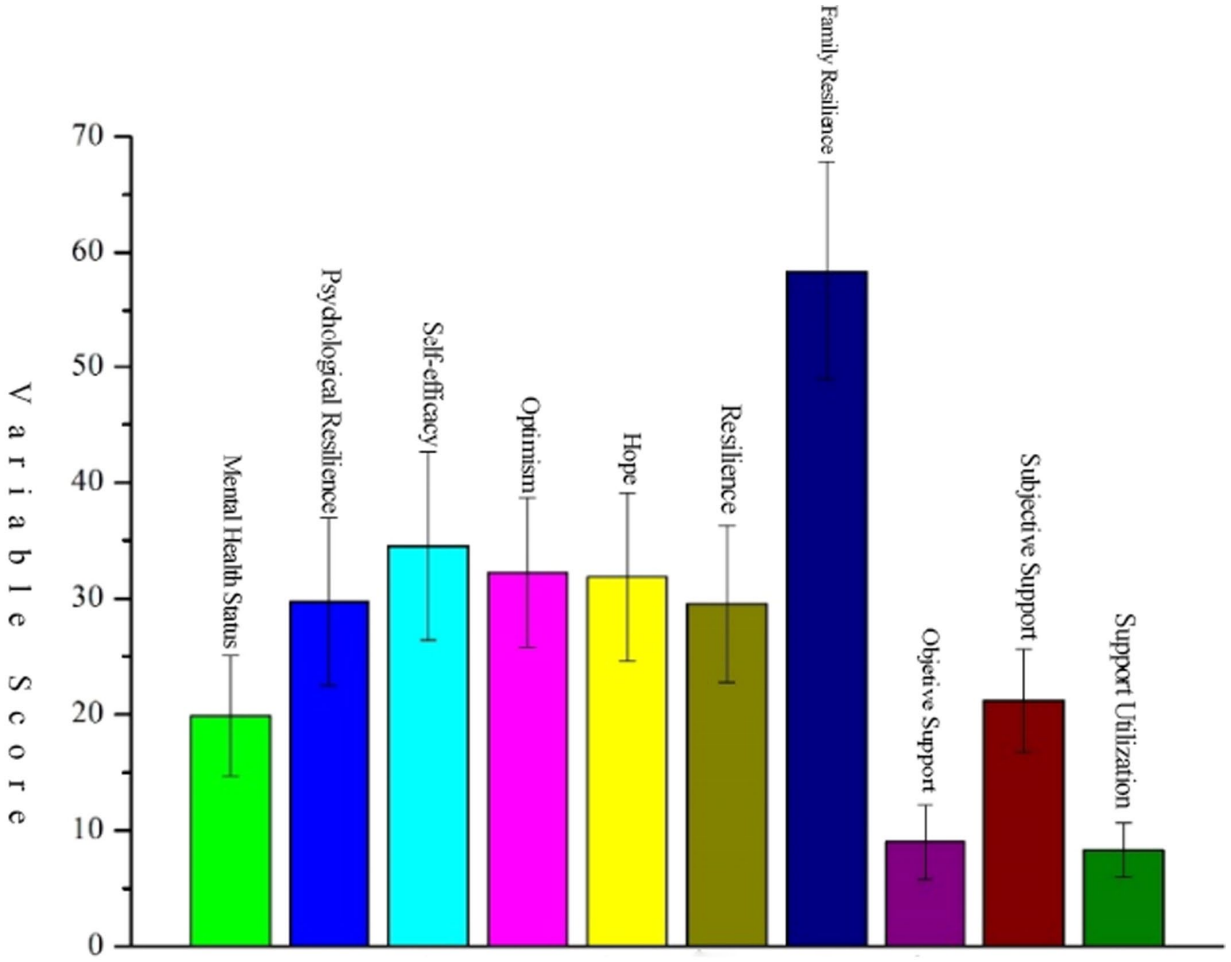
Score of variables related to psychological resilience, psychological capital, family resilience, social support, and mental health.

The correlation analysis indicates a substantial association between the mental health scores of university students and the scores of nine factors, encompassing psychological resilience, self-efficacy, optimism, hope, resilience, family resilience, objective support, subjective support, and support utilization (*p* < 0.01). Details are illustrated in Figures 2, 3.

### 3.3 Multivariate linear regression analysis of university students’ mental health

Taking the mental health score of university students as the dependent variable, this study considered demographic univariate analysis (grade and family economic status) and the nine factors with statistical significance from correlation analysis as variables. The inclusion criteria for the regression equation were set at 0.05, with 0.10 as the exclusion standard. In the final model, grade, family economic status, psychological resilience, self-efficacy, optimism, family resilience, objective support, and support utilization were included in the regression equation. These factors significantly predict university students’ mental health (*p* < 0.001) and collectively explain 57.9% of the total variation, details as shown in Table 2. The multivariate linear regression equation is presented in Eq. 1.


(1) $$\begin{array}{c} Y=0.226X_{1}-0.121X_{2}-0.363X_{3}-1.733X_{4}-0.112X_{5}\\ -0.119X_{6}-1.328X_{7}-2.127X_{8}+\varepsilon \end{array}$$

**Table 2:** Multivariate regression analysis with mental health status score as the dependent variable (*n* = 3,390).

Symbol	Variable	*B*	*SE*	*β*	*t*	*p*
*X*_1_	Grade	0.226	0.051	0.026	5.250	<0.001
*X*_2_	Economic status	−0.121	0.053	−0.010	−4.221	<0.001
*X*_3_	Psychological resilience	−0.363	0.076	−0.033	−8.133	<0.001
*X*_4_	Self-efficacy	−1.733	0.610	−0.786	−4.317	<0.001
*X*_5_	Optimism	−0.112	0.044	−0.018	−6.101	<0.001
*X*_6_	Family resilience	−0.119	0.029	−0.006	−10.866	<0.001
*X*_7_	Objective support	−1.328	0.543	−0.772	−4.418	<0.001
*X*_8_	Support utilization	−2.127	0.313	−0.672	−7.641	<0.001

*R*^2^ = 0.582, Adjusted *R*^2^ = 0.579, *F* = 29.137, *p* < 0.001.

The results of the multivariate linear regression analysis reveal a significant regression equation (*F* = 29.137, *p* < 0.001). Specifically, economic status (*β* = −0.010, *t* = −4.221, *p* < 0.001), psychological resilience (*β* = −0.033, *t* = −8.133, *p* < 0.001), self-efficacy (*β* = −0.786, *t* = −4.317, *p* < 0.001), optimism (*β* = −0.018, *t* = −6.101, *p* < 0.001), family resilience (*β* = −0.006, *t* = −10.866, *p* < 0.001), objective support (*β* = −0.772, *t* = −4.418, *p* < 0.001), and support utilization (*β* = −0.672, *t* = −7.641, *p* < 0.001) all significantly negatively impact the mental health status scores of university students. On the other hand, grade (*β* = 0.026, *t* = 5.250, *p* < 0.001) significantly positively influences the mental health status scores of university students. These independent variables collectively account for 57.9% of the variance in the dependent variable, the mental health status scores of university students.

## 4 Discussion

### 4.1 Disparities in the mental health status of Chinese university students

#### 4.1.1 Worse mental health status among graduating students

From Table 1, a substantial significant difference can be seen in the mental health status scores among university students across four grades, with graduating students exhibiting significantly poorer mental health conditions compared to those in the other three grades (*F* = 14.722, *p* < 0.01). This discrepancy can be attributed to several factors. Firstly, graduating students face immense pressure related to graduation. Research has consistently shown that pre-employment anxiety is prevalent among graduating students, and the prospects of securing employment directly impact the realization of individual career goals (22–25). The economic downturn caused by COVID-19 pandemic in the recent three years, with industries developed slowly like catering, entertainment, tourism, and international trade in particular, has led to a decrease in job opportunities and increased pressure on graduating students (26). With a record-high 11.79 million Chinese university graduates in 2024, the employment landscape poses considerable challenges, including limited job creation, a large pool of job seekers, and heightened anxiety in the digital age, contributing to the poorer mental health status of graduating students.

#### 4.1.2 Worse mental health status among economically disadvantaged students

Table 1 reveals a notable disparity in the mental health conditions of economically disadvantaged students when compared to their non-economically disadvantaged counterparts, showcasing a statistically significant difference between the two groups (*t* = 8.527, *p* < 0.01). The challenges faced by financially disadvantaged students may manifest in heightened economic, employment, and graduation pressures (27). These individuals often find themselves in need of stable employment to address immediate financial difficulties, but the process of job seeking, both online and offline, introduces tangible challenges which include but are not limited to the necessity of purchasing computers and covering essential transportation expenses. The harsh economic conditions compounded by uncertain job prospects contribute to hindrances in realizing employment goals, and declining demand for corporate recruitment fosters increased feelings of employment pressure and anxiety among economically disadvantaged students.

### 4.2 Dynamic system model of psychological resilience in mental health of university students

#### 4.2.1 Psychological resilience and positive psychological capital as facilitators of mental health of university students

In Figure 2, the scores of psychological resilience and positive psychological capital, including self-efficacy, optimism, hope, and resilience, demonstrate a significant negative correlation with the mental health status of university students (*p* < 0.01). The findings from regression analysis presented in Table 2 confirm that psychological resilience and components of positive psychological capital, particularly self-efficacy and optimism, significantly predict university students’ mental health (*p* < 0.001). Psychological resilience, reflecting an individual adaptability, optimism, and strength, plays a pivotal role in integrating resilience and strength factors during significant stress or setbacks. This focus facilitates goal-setting, decision-making, and positive coping with post-stress psychological states (28). Positive psychological capital refers to the positive psychological states exhibited by individuals during their growth and development processes. It encompasses four dimensions: self-efficacy, resilience, optimism, and hope. Additionally, high self-efficacy, a crucial element of positive psychological states, empowers individuals to regulate interpersonal relationships and manage social anxiety, thereby mitigating adverse effects of life events through positive cognitive and behavioral changes (29). The stable components of positive psychological capital, namely optimism, hope, and resilience, serve to alleviate psychological stress. Individuals possessing these psychological qualities maintain positive expectations during major crises, engage in positive behaviors to overcome conflicts and anxiety, and adapt to environmental changes, effectively coping with stress.

#### 4.2.2 Enhancing family resilience as an effective approach to improving mental health of university students

Family resilience specifically refers to the ability of the family system’s resources to adapt well to stress, serving as a characteristic or attribute of the family’s resilience. It acts as a family’s resilience factor, mediating the relationship between stress and mental illness (30). The analysis of Figures 2, 3 shows significant negative correlation between family resilience scores and university students’ mental health status (*p* < 0.01). The regression analysis presented in Table 2 affirms that family resilience serves as a robust predictor of university students’ mental health ( *p* < 0.001). The resilient belief, as a individual control factor of family resilience, maintains the conviction that positive actions can bring about change in the face of unforeseen events or disasters, leading to the alleviation of sadness or adversity (31). The challenge factor in family resilience encourages individuals to fully engage in stressful situations, seeking both internal and external support in stressful environment (32). The responsibility factor in family resilience functioning as an emotional bond among family members, fosters active commitment and mutual support. It acts as a preventive force against family breakdown during major disasters, and facilitates shared coping mechanisms in the face of difficulties. Moreover, it serves as a source of strength for self-growth during significant stress events.

#### 4.2.3 Positive social support: a protective factor for mental health of university students

Social support, as a protective factor for mental health (33), encompasses material and emotional sustenance adjusting individual stress derived from external sources, including family, friends, and colleagues (34). The affirmative impact of social support lies in its ability to assuage the detrimental effects of stress events, facilitating superior adaptation to environmental changes and a notable reduction in negative emotions (35). Figures 2, 3 intricately unveil a substantial negative correlation between scores of objective support, subjective support, support utilization, and university students’ mental health status (*p* < 0.01). A rigorous regression analysis, as detailed in Table 2，establishes the predictive power of objective support and support utilization in determining university students’ mental health (*p* < 0.001). Robust social support can indeed reduce negative stimuli in various aspects of life, including academics and daily living, thereby promoting mental health. In the realm of prolonged home-based living and academic study, if university students are endowed with substantial emotional support from family members, assistance from classmates and school teachers, and sustained interaction with close friends, they are poised to experience heightened emotional stability. This, in turn, fosters emotions, intimacy, adaptability, care, and confidence in various facets of life, contributing to augmented emotional stability, heightened psychological adaptability, and improved mental health levels among university students.

## 5 Recommendations

The aforementioned research results, based on the Psychological Resilience Dynamic System Model, indicate that the influencing factors on the mental health of university students encompass four main aspects. These include individual demographic factors such as grade and family economic status, positive psychological capital factors such as psychological resilience, self-efficacy, optimism, hope, and resilience, family resilience factors including responsibility, control, and challenge, and societal support factors including objective support, subjective support, and support utilization. On the basis of these findings, this paper proposes strategies and recommendations from four dimensions: universities, families, society, and individual university students.

### 5.1 Leverage the dominant role of university mental health education and stress intervention

Firstly, tailoring intervention for special groups is needed. Addressing the poorer mental health conditions of graduating students necessitates timely psychological counseling, graduation assistance, and employment guidance. Initiatives such as thesis guidance, building job platforms, and providing psychological counseling services are recommended. In response to economically disadvantaged students experiencing heightened mental health challenges, universities are advised to promptly provide economic assistance for graduating students in need. Secondly, universities should further make good use of virtual platforms for mental health education. It is vital to strengthen mental health education for all students through online courses and classroom moral education. Disseminating positive psychology concepts and disaster stress coping strategies can be achieved through a dual approach of “online courses” and “classroom moral education.” Thirdly, specialized and regularized Courses should be established. Universities are encouraged to promptly introduce specialized mental health courses related to coping with public crises and major emergencies. These courses can be incorporated into mandatory courses or integrated into physical education classes, effectively enhancing students’ capabilities in coping with sudden events and public crises.

### 5.2 Reinforce positive family philosophy and the educational influence of role models

Firstly, promoting a positive family educational philosophy is recommended. Cultivating this philosophy, which emphasizes harmonious relationships, responsibility, control, and challenge within families, contributes to elevating family resilience and enhancing the mental health levels of university students. Secondly, it is crucial to construct positive family educational approaches. A positive family education approach can help children gain a positive mindset and strengthen their personal resilience in times of major crises (36). Embracing modern education methods, families should utilize online tools such as QQ, WeChat, and Weibo to engage in equal and interactive communication with their children, being attentive to psychological changes during dialogues. Thirdly, Parents should act as role models. Parents serving as positive role models can guide their children effectively, setting a good example of self-discipline, composed behavior, and active involvement in family and social responsibilities, which will inspire positive emotions in children with courage and strength to make contribution to society.

### 5.3 Build a positive societal atmosphere and establish a support system for psychological counseling

Firstly, recognize the influential role of media as a positive influencer. An abundance of negative information, particularly for the general public, including university students, has the potential to evoke feelings of helplessness and disappointment. Such an environment is counterproductive to the cultivation of positive behaviors and psychological well-being. Therefore, it is imperative for news media to actively to create supportive societal atmosphere. They should interpret policy information in a timely, accurate and transparent manner, address public concerns with a positive outlook, and foster a media environment that nurtures confidence, unity, and warmth among the public. Secondly, emphasize the significance of counseling services. It is imperative to integrate and establish various psychological service assistance platforms across society during major emergencies. Timely dissemination of helpline information ensures those who have apparent psychological issues receive professional counseling, alleviating negative emotions and stress. Thirdly, recognize the educational role of society. Major crises can serve as profound educational experiences, and it is crucial to integrate mental health education and crisis education into daily life. This can be achieved, especially through voluntary services and other social practices, enabling students to experience positive emotions, aiding stress regulation, and enhancing the effectiveness of mental health education.

### 5.4 Empower university students through self-formation of psychological resilience and positive psychological capital

Firstly, emphasize positive self-cognition. In the context of significant disasters or adversities, insufficient self-awareness can readily contribute to a downturn or a feeling of being overwhelmed. When confronted with major unforeseen events, university students are encouraged to develop positive cognition by actively exploring their individual resources and strengths. It is essential for them to embrace self-affirmation, not only in themselves but also in their surrounding environment, which enhances psychological resilience and foster a greater sense of self-efficacy. Secondly, focus on positive coping strategies. Proactive coping strategies can yield positive outcomes (37). University students, when confronted with societal events such as the COVID-19 pandemic, should take proactive measures for disease prevention, actively engage in online knowledge acquisition, and participate enthusiastically in feasible social services. Thirdly, emphasize cultivating positive psychological capital. Positive psychological capital is cultivated progressively through postnatal efforts. University students consciously develop good habits, foster resilient determination by overcoming challenges, enhance self-control through rigorous demands, mitigate the adverse effects of unforeseen events through practical actions, and cultivate positive psychological qualities such as optimism, hope, and resilience. Through active practice, students elevate themselves, learn to take responsibility in unfavorable social environments, so as to contribute to national development and societal service.

## 6 Innovativeness and research significance

From the perspective of positive psychology, this study systematically introduces the Psychological Resilience Dynamic System Model as a theoretical framework into the unique population of Chinese university students in the post-pandemic era. Starting from internal and external protective factors, it explores the impact of psychological resilience, individual trait system as internal protective factors, family resilience as an external protective factor, and social support on the mental health of college students. By investigating the influencing factors on the mental health status of Chinese university students in the post-pandemic era, this study identifies protective factors for college students’ mental health. It proposes strategies and recommendations from four perspectives: universities, families, society, and college students themselves, providing theoretical foundations and personalized practical guidance for the development of mental health intervention measures for college students. This research holds significant theoretical implications and practical value.

### 6.1 Limitations and future directions

This study solely focused on university students from Zhejiang Province. With Zhejiang’s higher education system reflecting a microcosm of national higher education, consisting of 60 undergraduate universities as of 2023, including prestigious institutions like Zhejiang University, as well as more ordinary and local institutions like Zhejiang University of Technology and Shaoxing University, the selected universities were chosen using stratified sampling considering geographical and disciplinary distributions, thus reasonably representing the level and standards of higher education nationwide. However, due to constraints in research duration, manpower, and funding, the study did not opt for a nationwide sample, resulting in potentially incomplete sample representation and conclusions that may possess certain limitations. Future research could aim to expand the sample scope. This study conducted correlation and multiple regression analyses among the internal protective factors (individual trait system) and external protective factors (family support and social support). In the next step, with a sufficiently large sample size, further analysis could investigate the mediating factors influencing the mental health status of college students. Additionally, interventions such as group counseling and psychological resilience training could be implemented to enhance students’ psychological resilience, enabling them to cope more flexibly with uncertainty, reduce negative psychological states, and promote overall well-being.

## Data availability statement

The raw data supporting the conclusions of this article will be made available by the authors, without undue reservation.

## Ethics statement

The studies involving humans were approved by Ethics Committee of Zhejiang University of Science and Technology. The studies were conducted in accordance with the local legislation and institutional requirements. The participants provided their written informed consent to participate in this study.

## Author contributions

JF: Conceptualization, Formal analysis, Funding acquisition, Investigation, Resources, Supervision, Writing – original draft, Writing – review & editing. YH: Data curation, Formal analysis, Investigation, Methodology, Visualization, Writing – original draft. FY: Data curation, Investigation, Methodology, Writing – review & editing. YC: Investigation, Methodology, Visualization, Writing – review & editing. JY: Data curation, Investigation, Methodology, Project administration, Supervision, Writing – original draft, Writing – review & editing.
